# Supplementary material for: LI-YOLOv8: Lightweight small target detection algorithm for remote sensing images that combines GSConv and PConv
Source: PLoS One. 2025 May 23;20(5):e0321026. doi: 10.1371/journal.pone.0321026 (PMC12101674; doi:10.1371/journal.pone.0321026)
Supplement: Attachment — Submitted filename: review.docx [file pone.0321026.s001.docx]

1. LI-YOLOv8 shows significant improvements on remote sensing datasets like RSOD and NWPU VHR-10, how does it perform on more diverse and challenging datasets that are not specifically focused on small object detection? Could there be a risk of overfitting the small object characteristics of these datasets?
2. How does LI-YOLOv8 perform on real-world, noisy images with significant occlusions, varying lighting, or seasonal changes (detecting small objects in aerial imagery from different times of day or weather conditions)?
3. The reduction in parameters and GFLOPs is a notable advantage, but how sensitive is the model to changes in hyperparameters like learning rate, batch size, or anchor box configuration? What effect do these parameters have on performance, especially in terms of detection accuracy for small objects?
4. How does the training time and convergence behavior of LI-YOLOv8 compared to other algorithms like YOLOv8 and YOLOv5? Are there any challenges in training this model on large-scale remote sensing datasets?
5. Below Table-6 rephrase the sentence ("YOLOv8n's metrics are close to those of YOLOv5n" "YOLOv8n's metrics are similar to those of YOLOv5n.")
6. While the ablation studies demonstrate improvements when adding different innovations (SPPF-R, C2fE, GP-Detect, Inner-Wise), can you provide more insights into how each innovation specifically addresses challenges in small object detection? Which innovation contributes the most to improving recall or precision?
7. The experiments are mainly conducted on datasets like RSOD, NWPU VHR-10, and TinyPerson, which focus on specific types of remote sensing imagery. How does LI-YOLOv8 perform on datasets from other domains, such as medical imaging or urban surveillance, where small object detection might have different characteristics?
8. Does the model's emphasis on small object detection affect its ability to detect large objects, especially when these appear in similar scenes? Could there be a trade-off where performance on larger objects (e.g., buildings, vehicles) is compromised?
9. In the visual comparison with YOLOv8, LI-YOLOv8 shows fewer false detections in complex backgrounds. However, was there any trade-off between false positives and false negatives in the cases with small targets or occlusions? How does the model handle ambiguous cases?
10. While mAP@0.5 and mAP@0.5:0.95 are widely used, could there be additional evaluation metrics or domain-specific metrics (F1 score for small objects or intersection over union for very small objects) that would give a clearer picture of the model’s real-world performance?
