# Supplementary material for: LI-YOLOv8: Lightweight small target detection algorithm for remote sensing images that combines GSConv and PConv
Source: PLoS One. 2025 May 23;20(5):e0321026. doi: 10.1371/journal.pone.0321026 (PMC12101674; doi:10.1371/journal.pone.0321026)
Supplement: Attachment — Submitted filename: Response to Reviewers .docx [file pone.0321026.s002.docx]

Response to Journal Requirements:

1. We have shared the code, and the address is: https://github.com/2470589561/LI-YOLOv8

2. In our paper, regarding：This research was funded by the National Natural Science Foundation of China (no. 62173171).

The funding statement needs to be revised to”The funders had no role in study design, data collection and analysis, decision to publish, or preparation of the manuscript.”

3. We will share the minimum dataset, the address is: https://github.com/2470589561/datasets

4. The 14th figure in the manuscript contains satellite images, which are from the publicly available geospatial object detection dataset NWPU VHR-10 for research purposes. The source of the data is: <https://gcheng-nwpu.github.io/#Datasets> , In order to respect the labor achievements and intellectual property rights of the data providers, the relevant papers have been cited in the manuscript. There is no copyright issue.

Dear reviewers, thank you for your comments. We have completed the revisions to the manuscript according to the reviewer's suggestions.

The response to reviewers is as follows：

Response to reviewer 1

1. Language and Clarity: Simplify the language to make sentences more concise. Some sections lack clear structure, such as overlapping content in the "Methods" and "Algorithm Implementation" sections. Redundant sentences, especially in the background introduction, affect readability. Overly detailed explanations of terms and formulas may confuse non-specialist readers.

Response: The content of the manuscript has been simplified in language and improved in terms of enhancing the coherence between sentences.

2. Use of Figures and Explanations: There is insufficient explanation and referencing of figures. For example, in figures such as Fig.11 and Fig.12, only the improvement in PR curves is mentioned without detailing the reasons for the changes and their impact on results. It is recommended to provide more analysis and interpretation of the figures in the text.

Response: Detailed supplementary explanations are provided in Figures 11(a), (b) and Figures 12(a), (b). These not only describe the degree of fluctuation in the PR curve and its causes, but also include a comparative analysis of the two PR graphs (a) and (b), as well as the effects achieved by the four improvement points in the model.

3. Description of GP-Detect Module: The explanation of how the GP-Detect module reduces redundant computations is somewhat superficial and lacks in-depth theoretical support.

Response: Supplementary description of GP-Detect.

4. Ablation Experiments: Provide more quantitative or qualitative discussions on the specific contributions of each module in the ablation experiments. For instance, what are the primary reasons behind the 1.2% improvement attributed to SPPF-R?

Response: In the ablation experiments, a quantitative and qualitative discussion of the specific contributions of each module has been added.

5. Broader Validation: Supplement the study by validating the proposed model and methodology on at least two additional datasets or datasets from related domains to demonstrate the model's generalizability and practical applicability.

Response: Based on the feedback from another reviewer regarding the differing performance of the algorithm in medical imaging and urban surveillance, three datasets (LEVIR-ship, brain-tumor, and smoke_fire_1) have been added in the generalization experiments section to validate the generality and generalization capability of LI-YOLOv8. The LEVIR-ship dataset is a small vessel dataset of remote sensing images, which verifies that the algorithm still has good recognition performance for small targets in the same domain. The brain-tumor dataset is used for detecting brain tumors in medical images, demonstrating the algorithm's versatility and indicating that it performs well with non-specific targets in other fields. The smoke_fire_1 dataset is used for detecting fire targets in urban surveillance, validating the algorithm's generality.

Response to reviewer 2

1. LI-YOLOv8 shows significant improvements on remote sensing datasets like RSOD and NWPU VHR-10, how does it perform on more diverse and challenging datasets that are not specifically focused on small object detection? Could there be a risk of overfitting the small object characteristics of these datasets?

Response: Based on your suggestion, the generalization experiments section has been updated to include the brain tumor detection dataset and the fire smoke monitoring dataset, both of which are non-small target datasets in the areas of medical imaging and urban surveillance. The experimental results indicate that LI-YOLOv8 performs well in detecting non-small targets, and there is no risk of overfitting.

2. How does LI-YOLOv8 perform on real-world, noisy images with significant occlusions, varying lighting, or seasonal changes (detecting small objects in aerial imagery from different times of day or weather conditions)?

Response: Our research focuses on small target detection in remote sensing images, which have their own unique characteristics. Remote sensing images are typically high-definition and high-resolution, leading to significant differences in processing these images compared to general noisy images. In remote sensing images, the likelihood of target occlusion is relatively low. Due to the high vantage point from which remote sensing images are captured, there is a reduced probability of targets blocking each other. Regarding lighting conditions, remote sensing images generally have stable lighting, as they are often acquired under specific conditions that provide relatively uniform illumination. Additionally, because remote sensing images cover a wide area, local variations in lighting have a minimal impact on the overall image. Therefore, our study does not include images from multiple time periods or different weather conditions (such as nighttime, rainy days, or snowy days).

3. The reduction in parameters and GFLOPs is a notable advantage, but how sensitive is the model to changes in hyperparameters like learning rate, batch size, or anchor box configuration? What effect do these parameters have on performance, especially in terms of detection accuracy for small objects?

Response: Firstly, regarding the anchor box configuration issue you mentioned. In fact, YOLOv8 is an anchor-free detection model. During the detection phase, the model does not rely on predefined anchor boxes to propose candidate object locations; the final object detection is directly based on the detected features. Therefore, in our research, there is no issue of anchor box configuration affecting the model. Secondly, the experimental environment for LI-YOLOv8 is a NVIDIA RTX3090 GPU with 24GB VRAM, 14 vCPUs of Intel(R) Xeon(R) Gold 6330 CPU @ 2.00GHz, and 80GB RAM. Ablation, comparison, and generalization experiments are conducted on different datasets and models. Model improvement and training must be carried out under the premise of the same configured hyperparameters and batch size. Setting hyperparameters is crucial for shaping model performance and ensuring the successful enhancement of the algorithm. In the process of refining the YOLOv8 model, it is essential to adhere to a unified hyperparameter configuration. This consistency is key; it confirms the effectiveness of model improvements and accelerates the precise performance evaluation conducted before and after the improvements. Adjusting hyperparameters during the algorithm refinement process may obscure the root causes of performance changes—whether they are due to inherent algorithm enhancements or the results of hyperparameter changes. Therefore, to determine a clear and rigorous assessment of progress, this paper firmly follows a set of standardized hyperparameters.

4. How does the training time and convergence behavior of LI-YOLOv8 compared to other algorithms like YOLOv8 and YOLOv5? Are there any challenges in training this model on large-scale remote sensing datasets?

Response: Thank you to the reviewer for your valuable comments. We have conducted a detailed analysis of the training time and convergence behavior of LI-YOLOv8:

(1) Training Time and Convergence Behavior:

Under the same hardware conditions (RTX 3090 with 24GB of video memory), the training time of LI-YOLOv8 is about 15% longer than that of YOLOv8n. This is mainly due to the addition of modules such as RFAConv and EMA attention mechanisms, which increase the computational load per iteration. However, from the analysis of convergence behavior, the loss value of LI-YOLOv8 is lower than that of YOLOv8n. This indicates that although the training time per round is slightly longer, the convergence is better.

(2) In the training of large-scale remote sensing datasets, there are two main challenges: First, the attention modules consume a large amount of video memory when processing high-resolution images. We alleviated this issue by optimizing the batch size and using mixed-precision training. Second, the features of small targets vary significantly across different scenes. By designing the Inner-Wise IoU loss function, we enhanced the model's ability to learn from samples of varying quality.

Although there is a slightly longer training time, considering the significant advantages of LI-YOLOv8 in detection accuracy and model lightweighting, we believe this is an acceptable trade-off. The convergence behavior of LI-YOLOv8 is better than that of YOLOv8n, but compared to other algorithms in the YOLO series, its convergence behavior is not the optimal choice. Future work will be dedicated to further optimizing training efficiency to make the model more suitable for large-scale practical applications. In the conclusion section, we have supplemented the expectations and future plans for the model, and further optimization of the model will be carried out in the future.

5. Below Table-6 rephrase the sentence ("YOLOv8n's metrics are close to those of YOLOv5n" "YOLOv8n's metrics are similar to those of YOLOv5n.")

Response: The sentence has been revised.

6. While the ablation studies demonstrate improvements when adding different innovations (SPPF-R, C2fE, GP-Detect, Inner-Wise), can you provide more insights into how each innovation specifically addresses challenges in small object detection? Which innovation contributes the most to improving recall or precision?

Response: The contributions of each innovation to the model have been added and described in the ablation experiment section.

7. The experiments are mainly conducted on datasets like RSOD, NWPU VHR-10 and TinyPerson, which focus on specific types of remote sensing imagery. How does LI-YOLOv8 perform on datasets from other domains, such as medical imaging or urban surveillance, where small object detection might have different characteristics?

Response: In the generalization experiment section, three datasets (LEVIR-ship, brain-tumor, and smoke_fire_1) were added to validate the universality and generalization capability of LI-YOLOv8. Among them, LEVIR-ship is a remote sensing image dataset for small vessels, validating the algorithm's recognition performance for small targets within the same domain. The brain-tumor dataset is for detecting brain tumors in medical images, demonstrating the algorithm's versatility and indicating that it performs well for non-specific targets in other fields. The smoke_fire_1 dataset is used for detecting fire targets in urban surveillance, further validating the algorithm's generality.

8. Does the model's emphasis on small object detection affect its ability to detect large objects, especially when these appear in similar scenes? Could there be a trade-off where performance on larger objects (e.g., buildings, vehicles) is compromised?

Response: The model's detection of small objects does not affect its ability to detect large objects. The RSOD dataset contains a large number of small objects, as well as some medium and large objects. LI-YOLOv8 has significantly improved the detection of all four types of objects in the RSOD dataset, which can prove this point. When designing the LI-YOLOv8 model, we did indeed place special emphasis on the detection capability of small objects. This is because in remote sensing images, the detection of small objects (such as small vehicles, boats, etc.) is often more challenging and is of great significance in many application scenarios. However, we also fully considered the detection performance of large objects (such as buildings, large vehicles, etc.) in the model design to ensure the balance of the model in detecting objects of different scales. At the same time, three datasets were added in the generalization experiments to verify the universality and versatility of the algorithm, indicating that the algorithm also has a good improvement in the detection effect of non-specific objects in other fields.

9. In the visual comparison with YOLOv8, LI-YOLOv8 shows fewer false detections in complex backgrounds. However, was there any trade-off between false positives and false negatives in the cases with small targets or occlusions? How does the model handle ambiguous cases?

Response: In situations involving small targets or occlusion, the design and optimization of LI-YOLOv8 indeed require a trade-off between false positives and false negatives. Our model addresses this trade-off by incorporating SPPF-R, C2f-E, GP-Detect, and Inner-Wise: SPPF-R enhances small target feature extraction, C2f increases the focus on small targets, GP-Detect provides a lightweight algorithm while maintaining model performance, and Inner-Wise improves generalization capability. The remote sensing image datasets consist of high-resolution images and do not exhibit significant blurriness. LI-YOLOv8 aims to improve the detection and accuracy of small targets as much as possible while remaining lightweight.

10. While mAP@0.5 and mAP@0.5:0.95 are widely used, could there be additional evaluation metrics or domain-specific metrics (F1 score for small objects or intersection over union for very small objects) that would give a clearer picture of the model’s real-world performance?

Response: F1-score has been introduced as an evaluation metric, and the F1-score experimental results have been added to Tables 1 and 2 in the ablation experiments to validate the model's overall performance.

Response to reviewer 3

1. The manuscript may require some improvement to the English.

Response: The content of the manuscript has been simplified in terms of language, and improvements have been made to enhance the coherence between sentences.

2. From the experimental results presented in Table 1, it can be seen that there is no significant improvement in mAP and other parameters before and after using Inner Wise IoU. Similarly, the improvements observed in Table 2 are also insignificant. Is the use of Inner-Wise IoU appropriate?

Response: From the overall experimental design and result analysis of the paper, it is true that the improvement of Inner-Wise IoU is relatively limited in Table 1 and Table 2. However, whether it is "appropriate" cannot be judged solely based on the single numerical increase.

Firstly, from the stability and robustness of boundary regression, even if some experimental tables show that the improvement of mAP after introducing Inner-Wise IoU is not significant, it may be helpful in the stability of location regression and the recognition of hard - to - detect samples. Our manuscript mentions that Inner-Wise IoU introduces a "dynamic non - monotonic focusing mechanism" and an "auxiliary bounding box" strategy, aiming to provide more reasonable gradient allocation for ordinary quality anchor boxes (not particularly high-quality or low-quality annotations), thereby further stabilizing the regression process in complex backgrounds or when the target scale varies. If we only look at the single mAP value, the increase may not be large; but when considering aspects such as positioning error or robustness performance, such improvements often have their value.

Secondly, the improvement may be "diluted" by other enhancements. The paper also introduces multiple modules such as SPPF-R, C2f-E, and GP-Detect. When multiple improvements are stacked together, sometimes there can be a phenomenon of "saturation value of increase" or "low contribution prominence": when the preceding several enhancements have significantly optimized feature extraction, attention mechanisms, and the lightweighting of detection heads, subsequent changes (such as the introduction of the Inner - Wise IoU function) are inevitably "diluted" by the high baseline performance of the overall algorithm, and numerically, there may not be very exaggerated changes. However, this does not mean that it has no positive effect, especially in generalization experiments (TinyPerson) or other complex small - target scenarios, where certain benefits may still be observed.

If the sole pursuit is to maximize the mAP metric on a specific dataset, and this dataset itself has high annotation accuracy and a relatively uniform distribution, then the improvement brought by Inner-Wise IoU may indeed be limited, and it could even be considered for simplification. However, if the project requirements focus on "robust detection of algorithms in various remote sensing scenarios and data with extremely unbalanced small-target distributions," and "taking into account annotations of different qualities and with a certain risk of confusion," then Inner-Wise IoU has research and application value that is worth retaining.

Taking into account the above points, although the benefit of Inner-Wise IoU does not seem particularly high in terms of surface numerical increase, it may still be helpful in aspects such as positioning stability and generalization ability. By supplementing the contribution of individual innovations to the algorithm in Table 1 and Table 2, Inner-Wise IoU remains a suitable choice from the perspective of experimental result values.

3. The manuscript mentioned that LI-YOLOv8 not only detects more small targets in complex backgrounds but also does not incur any false detections.However, the detection accuracy of the vehicle in Table 4 has decreased by 5.2%. Does this contradict the conclusion of the manuscript？

Response: Thank you for pointing out the deficiencies. The manuscript has been revised regarding the parts where no false detections occurred. The design of LI-YOLOv8 in the manuscript aims to enhance the mean average precision (mAP) of small objects across all categories on the basis of model lightweighting. Although the vehicle category, which occupies a small proportion in images, has a poor learning ability during training, leading to a decrease in detection accuracy, considering the significant advantages of LI-YOLOv8 in terms of overall mAP and model lightweighting, we believe this is an acceptable trade-off. Moreover, the LI-YOLOv8 algorithm has been tested for generalization on multiple related and different domain datasets, and the experimental results reflect the universality and versatility of the LI-YOLOv8 algorithm. In the future, we expect to further strengthen the research on improving the detection of small vehicle objects.

4. Tables 5 and 6 seem to be simple stacks of algorithms. Please explain the significance of comparing YOLO algorithms at different stages and versions?

Response: In Tables 5 and 6, we conducted experimental comparisons between the proposed lightweight improved version (LI-YOLOv8) and various existing YOLO series algorithms (such as YOLOv3-tiny, YOLOv5n/s, YOLOv6s, YOLOv8n, YOLOv9c, YOLOv10n, etc.). The reason for choosing to compare with YOLO algorithms at different stages and versions is based on the following points:

(1) The YOLO series algorithms have undergone continuous optimization and evolution from YOLOv3, YOLOv4, and YOLOv5 to YOLOv6, YOLOv7, YOLOv8, YOLOv9, and YOLOv10, in aspects such as network structure, activation functions, loss functions, and feature fusion methods. Each version represents certain characteristics under different hardware configurations, dataset features, and detection scenarios. Comparing these versions can more comprehensively reflect the relative advantages or shortcomings of the proposed algorithm within different generations of the YOLO framework.

(2) In small target detection scenarios, it is typically necessary to balance a high detection rate, good accuracy, and low parameter count and computational load. The YOLO series of algorithms has evolved from an initial focus on detection speed to a later emphasis on balancing parameter size, accuracy, and speed. Each version makes different trade-offs between 'lightweight design and performance.' Comparing LI-YOLOv8 with various versions allows readers to better understand the differences in accuracy and lightweight design between LI-YOLOv8 and larger parameter algorithms (such as YOLOv9c, YOLOv10n), as well as the differences in speed and accuracy compared to 'simplified/lightweight' versions (such as YOLOv3-tiny, YOLOv5n).

In summary, different stages and versions of YOLO algorithms each have their own innovative highlights, applicable scenarios, and performance focuses. Comparing them with the improved algorithm proposed in this study in the same context allows for a comprehensive evaluation of the advantages and improvements achieved in small target detection through various metrics (mAP, Precision, Recall, Params, GFLOPs, etc.). This also enhances the comparative significance and persuasiveness of the experimental results presented in the paper.

5. Line502-Line504: The previous research compared LI-YOLOv8 with YOLO series algorithms, and this paragraph also compared it with R-cnn. What does the author want to express? The logic of the manuscript is a bit confusing.

Response: The generalization experiments of the R-CNN algorithm have been removed. Following the suggestions provided by other reviewers, three datasets (LEVIR-ship, brain-tumor, and smoke_fire_1) have been added to the generalization experiment section to verify the versatility and generalization ability of LI-YOLOv8. This demonstrates that LI-YOLOv8 not only performs well in small object detection of remote sensing images, but also shows good performance in non-small object categories in other fields such as medical imaging and urban surveillance.
